# MiR‐155 promotes colitis‐associated intestinal fibrosis by targeting HBP1/Wnt/β‐catenin signalling pathway

**DOI:** 10.1111/jcmm.16445

**Published:** 2021-03-26

**Authors:** Nianshuang Li, Yaobin Ouyang, Xinbo Xu, Zhenxiang Yuan, Chunquan Liu, Zhenhua Zhu

**Affiliations:** ^1^ Department of Gastroenterology The First Affiliated Hospital of Nanchang University Nanchang China; ^2^ Institute of Digestive Disease The First Affiliated Hospital of Nanchang University Nanchang China

**Keywords:** Crohn's disease, HBP1, intestinal fibrosis, miR‐155, Wnt/β‐catenin signalling pathway

## Abstract

Intestinal fibrosis is the most common complication of Crohn's disease (CD) that is one major disorder of inflammatory bowel disease (IBD), but the precise mechanism remains unclear. MiR‐155 has been involved in fibrotic diseases. Here, we determined the role of miR‐155 in regulating intestinal fibrosis. MiR‐155 levels were significantly up‐regulated in CD patients with intestinal stricture CD. The overexpression of miR‐155 significantly aggravated TNBS‐induced CD‐associated intestinal fibrosis. Mechanistically, we identified that HBP1, a negative regulator of the Wnt/β‐catenin signalling pathway, is a direct target of miR‐155. Moreover, in vitro and in vivo experiments suggested that the miR‐155/HBP1 axis activates Wnt/β‐catenin signalling pathway to induce intestinal fibrosis. Taken together, we demonstrated that miR‐155 directly targets HBP1 to induce CD‐associated intestinal fibrosis via Wnt/β‐catenin signalling pathway.

## 1 INTRODUCTION

Crohn's disease (CD) is a type of inflammatory bowel disease (IBD) that causes chronic inflammation of the gastrointestinal tract. The prevalence of CD appears to be increased in recent years.^1^, ^2^ Even though the patients present with the inflammatory disease only, long‐term inflammation often results in bowel stricture and fibrotic lesions.^3^, ^4^ After the initial injury and inflammatory phase, efficient wound healing and tissue repair take place to return to normal physiological homeostasis. However, aberrant wound healing resulting from persistent inflammation causes excessive deposition of the extracellular matrix (ECM), which is promoted by the expansion of the fibroblast and myofibroblast cells.^4^, ^5^, ^6^ Currently, there is no effective therapeutic strategy to prevent fibrosis development in CD patients.^7^ A better understanding of the mechanism of intestinal fibrosis will improve radically to provide a potential treatment for CD patients.

MiRNA is a class of small 18‐25 nucleotide non‐coding RNAs that regulate target gene expression by degradation or translational repression.^8^ It has been demonstrated that miRNAs play a crucial role in a wide range of biological processes, such as cell proliferation, cell cycle, apoptosis and cell differentiation.^9^, ^10^ MiR‐155 is one of the best‐characterized miRNAs that is encoded by the host gene MIRHG155, initially identified as the B‐cell Integration Cluster (BIC) gene.^11^ The aberrant expression of miR‐155 has been found in various tumour types, such as lung cancer,^12^ colon cancer^13^ and cervical cancer.^14^ Moreover, it is also well known that the dysregulation of miR‐155 is involved in the immune and inflammatory cell response.^15^ Fibrosis is a pathological state of low‐grade chronic systemic inflammation. Wang et al indicated that miR‐155‐enriched in exosomes could inhibit cardiac fibroblast proliferation by suppressing Son of Sevenless 1 (Sos1) protein and induced inflammation by regulation of Socs1/Stat3 signalling.^16^ MiR‐155 has been demonstrated to regulate the development of liver fibrosis and alcoholic liver disease. Knockout of miR‐155 in mice significantly attenuates CCl4‐induced liver fibrosis, alcohol‐induced liver inflammation and injury.^17^ These data suggest that miR‐155 could be a potential therapeutic target for fibrotic disease. However, the functional role of miR‐155 in CD‐associated intestinal fibrosis remains unknown.

The canonical Wnt/β‐catenin pathway is critical for several biological processes during embryonic development and tissue homeostasis.^18^ In the ‘Wnt‐off’ state, the destruction complex composed of β‐catenin, APC, GSK3β and CK1‐α triggers the cytoplasmic accumulation of β‐catenin. In the ‘Wnt‐on’ state, the inactivation of destruction complex results in the increased β‐catenin levels and its nuclear accumulation. Activated β‐catenin then interacts with TCF/LEF family members and induces downstream gene expression.^19^, ^20^ The dysregulation of Wnt/β‐catenin signalling pathway has been implicated in the progression of various fibrotic diseases. β‐catenin was frequently dysregulated in the development of fibrotic disease. Increased expression of β‐catenin was frequently reported in human liver fibrotic tissues. Activation of β‐catenin could promote collagen production and proliferation of hepatic stellate cells.^21^ It has been found that hyperactivation of the Wnt/β‐catenin pathway is associated with renal fibrosis in chronic kidney disease.^22^, ^23^ Nevertheless, the role of Wnt/β‐catenin signalling pathway in colitis‐associated intestinal fibrosis needs to be clarified.

The HMG‐box transcription factor HBP1 is identified as a tumour suppressor that negatively regulates the cell cycle.^24^ On one hand, HBP1 is known as a negative regulator of β‐catenin transactivation. The investigation in tumour cells indicated that HBP1 efficiently regulates Wnt signalling pathway to inhibit cell proliferation through the suppression of TCF‐β‐catenin complex.^25^ It has been reported that the hypermethylation of HBP1 promoter is responsible for the loss of HBP1 in human non‐small cell lung cancer (NSCLC), which results in the poor prognosis of cancer through promoting β‐catenin activity.^26^ On the other hand, recent studies have confirmed that HBP1 is the direct target of miR‐155. Tian et al^27^ found that miR‐155 regulates lipid uptake and ROS production of macrophages in atherosclerotic development by direct repression of HBP1. Also, miR‐155 has been demonstrated to promote the proliferation and cell growth of osteosarcoma through targeting HBP1.^28^ However, whether miR‐155 is involved in the progression of CD‐associated intestinal fibrosis by mediating HBP1 and Wnt signalling has not been reported.

In this study, we found that the up‐regulation of miR‐155 induces intestinal fibrosis by targeting HBP1. Furthermore, we found that miR‐155 regulates colitis‐associated intestinal fibrosis by induction of the Wnt/β‐catenin signalling pathway. The overexpression of miR‐155 in TNBS‐induced colitis in mice significantly exacerbates intestinal fibrosis, whereas inhibition of miR‐155 resulted in the opposite effect. In patients with severe stricture CD, miR‐155 levels were significantly elevated. These results reveal a new regulatory mechanism that miR‐155/HBP1 axis promotes colitis‐associated intestinal fibrosis via Wnt/β‐catenin signalling pathway and suggest that miR‐155 is a potential therapeutic target for CD treatment.

## 2 MATERIALS AND METHODS

### 2.1 Cell culture

Human colon‐derived CCD‐18Co myofibroblast cells and human embryonic kidney 293T (HEK293T) cells were used in this study. Cells were purchased from American Type Culture Collection (ATCC) and cultured using DMEM medium (HyClone) supplemented with 10% of foetal bovine serum (FBS) and 1% penicillin/streptomycin solution (Invitrogen). Cells were maintained at 37°C in 5% CO_2_ atmosphere and passaged every 2 days.

### 2.2 Human tissue samples

A total of 49 human colonic mucosa samples were from endoscopic patients were collected, which included 25 of non‐stricture CD (NSCD) and 24 of stricture CD (SCD). Additional 22 human colonic mucosa biopsies were collected from healthy patients. Samples were obtained when patients were undergoing colonoscopy in the Department of Gastroenterology of the First Affiliated Hospital of Nanchang University. The diagnosis of NSCD and SCD was confirmed by clinical and pathological diagnosis. All specimens were frozen immediately in liquid nitrogen after endoscopy to detect the level of miR‐155 and HBP1. The study protocol and informed consent were approved by the Ethics Committee of The First Affiliated Hospital of Nanchang University.

### 2.3 Animals

Six‐ to eight‐week‐old female BALB/C mice (specific pathogen‐free, 20‐25 g weight) were divided into 5 groups (n = 6 for each group) including control group, CD group, CD+ miR‐NC group, CD+ miR‐155 mimic group and CD+ miR‐155 inhibitor group. The CD animal model was induced by TNBS (Sigma‐Aldrich).^29^ Briefly, following fasting for 24 hours, a rubber catheter was inserted into the rectum of mice. Animals were injected with 0.1 mL of TNBS (2 mg in 50% ethanol), once a week for 7 weeks. Before building the CD animal model, miR‐NC, miR155 mimics or miR155 inhibitor (RiboBio) were injected intravenously through the tail vein of colitis group mice at a dosage of 5 × 10^9^ pfu one day. After the end of the last injection of TNBS for 1 week, mice were killed, and colonic tissues were harvested. All procedures were in accordance with The Ethics Committee of the First Affiliated Hospital of Nanchang University.

### 2.4 H&E and Masson staining

Formalin‐fixed and paraffin‐embedded mice colonic specimens were sectioned at 4 μm thickness. The sections were baked at 60°C for 3 hours, de‐paraffinized in xylene and rehydrated with gradient ethanol. Then, H&E staining was used to evaluate the colon histopathologic appearance. In addition, the distribution and amount of connective tissue was assessed by Masson's trichrome staining (Masson's staining) according to the manufacturer's instructions.

### 2.5 Immunofluorescence

Immunofluorescence staining was previously described.^30^ Briefly, after rehydrated with gradient ethanol, sections were repaired with EDTA solution at 100°C for 15 minutes and then washed by PBS solution 3 times. Non‐specific binding sites were blocked by incubation with goat serum at room temperature for 30 minutes. After blocking, sections were incubated with the primary antibodies overnight at 4°C and with a secondary fluorescent antibody for 1 hour at 37°C, respectively. Then, sections were counter‐stained with DAPI (Beyotime Biotechnology) and evaluated by a fluorescent microscope (Olympus bx53 biomicroscope). The following primary antibodies were used: α‐SMA antibody (Proteintech), Collagen Type I (Collagen I) antibody (Proteintech) and β‐Catenin antibody (Proteintech).

### 2.6 miRNA 155 target prediction

A web‐tool was used for the prediction of miRNA155 targets (TargetScan, http://www.targetscan.org/vert_70/). Raw data were manually screened, and the selected target was further validated.

### 2.7 Transient transfection

Transfection of miR‐155 mimics, inhibitors (RiboBio) and siRNAs (RiboBio) were performed with Lipofectamine 2000 (Invitrogen) according to the manufacturer's instructions. The most effective siRNA identified by qRT‐PCR was used for subsequent experiments. After transfection, cells were harvested for Western blot and qRT‐PCR analyses. The primers for siRNAs were listed in Table S1.

### 2.8 Western blotting

The colonic tissue and cell samples were homogenized in RIPA lysis buffer (Beyotime Biotechnology) supplemented with 1 mmol/L PMSF, then centrifuged at 12 000 *g* for 10 minutes and total proteins in the supernatant were collected. The extraction of nuclear and cytoplasmic proteins was prepared according to the instructions of a nuclear and cytoplasmic extraction kit (BestBio). Total proteins were separated by SDS‐PAGE gels, which were prepared depending on the protein size and the separated and proteins were then transferred to a PVDF membrane. Electrophoresis and transmembrane were carried out on protein electrophoresis and blotting system (Liuyi). After the membranes were blocked with 5% non‐fat milk at room temperature for 1 hour, detection of importin binding was performed by incubating blots with primary antibody at 4°C overnight and with secondary antibodies at room temperature for 1 hour, respectively. Blots were visualized using the Immun‐Star Western Chemiluminescence Kit (Liuyi), and images were captured using a ChemiDoc XRS + System and processed using Image Lab software (Liuyi). Antibodies used in the described study were listed in Table S2, and GAPDH was used as an internal reference for cytoplasmic/total proteins and Lamin B for nuclear proteins.

### 2.9 qRT‐PCR assays

Total RNA was extracted from colonic tissue and cell sample using TRIzol reagent (Invitrogen) and converted to cDNA using the reverse transcriptase kit (Vazyme) according to the manufacturer's protocol. Amplification was performed on a real‐time PCR system (Applied Biosystems 7500). Transcript levels of targeted genes were assayed. The whole amplification procedure was according to the manual of SYBR® Premix Ex Taq™ kit (Takara RR420A). Relative expression was calculated using the formula of $2^{‐\Delta \Delta C_{T}}$. The primers for detecting gene expression were shown in Table S3. GAPDH or U6 was used as an internal reference.

### 2.10 Luciferase reporter assays

The HBP1‐3’UTR‐wt was purchased from company (Tianyihuiyuan Biotechnology) and was subcloned into pYr‐MirTarget luciferase vector using NotI and XhoI restriction sites. The mutant was obtained by QuikChange^®^ mutagenesis (Themo Fisher). For reporter assays, the WT or MT vector was co‐transfected into HK293T cells with miR‐155 mimics, miR‐155 mimics inhibitors. The cells were then harvested for luciferase assay by Dual‐Luciferase® Reporter Assay Systems (Promega Corporation) as described previously (see the Supplementary Experimental section at http://www.biochemj.org/bj/460/bj4600025add.htm).

### 2.11 Statistical analysis

All data are presented as mean ± standard deviation (SD). Statistical analysis was performed by SPSS18.0 software using Mann‐Whitney, Student's *t* test and one‐way analysis of variance (ANOVA) based on the data set. Statistical significance was indicated at *P < 0.05, **P < 0.01 or ***P < 0.001.

## 3 RESULTS

### 3.1 MiR‐155 is elevated in CD patients with intestinal stricture

**FIGURE 1:**
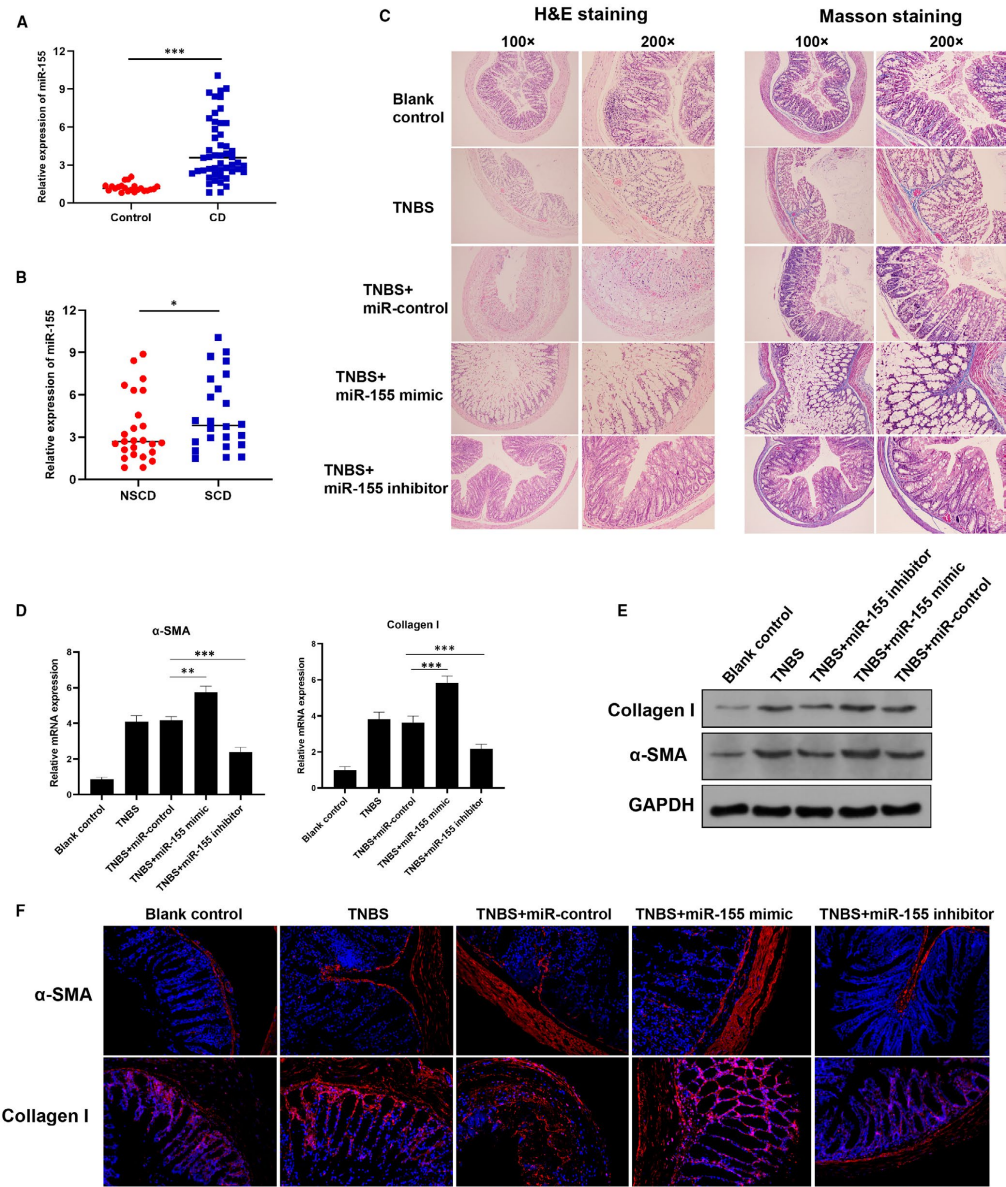
MiR‐155 is overexpressed in the patients with stricture CD and promoted intestinal fibrosis in the TNBS‐induced colitis mouse model. A, qRT‐PCR analysis for miR‐155 expression was performed in CD patients and healthy controls. B, MiR‐155 expression was further compared in CD patients with or without intestinal stricture. NSCD, non‐stricture CD; SCD, stricture CD. C, BABL/C mice were treated with TNBS to induce colitis and then administrated with miR‐155 mimic, inhibitor or control adenovirus. Intestinal fibrosis was detected by H&E staining or Masson staining. The representative images were shown (Magnification 100× or 200×). D, The mRNA levels of fibrosis markers including α‐SMA and Collagen I in each group by qRT‐PCR analysis. E, The protein expression of fibrosis markers by Western blot analysis. F, Expression and localization of fibrosis markers including α‐SMA (Red) and Collagen I (Red) were visualized by immunofluorescence. The blue‐fluorescent DAPI was used for nuclear staining (Magnification 200×). N = 6. Data expressed as mean ± SD. *P < 0.05, **P < 0.01, ***P < 0.001

To identify the role of miR‐155 in CD progression, we firstly detected the expression of miR‐155 in CD patients by qRT‐PCR assay. The results indicated that miR‐155 expression was significantly elevated in CD patients compared with healthy controls (Figure 1A). Furthermore, the levels of miR‐155 were analysed in CD patients with or without intestinal stricture. Interestingly, miR‐155 was significantly elevated in CD patients with intestinal stricture, compared to that patients without stricture (Figure 1B). These data suggest that miR‐155 is involved in the progression of CD with an intestinal stricture.

### 3.2 MiR‐155 promoted intestinal fibrosis in TNBS‐induced colitis mouse model

Stricture formation, as the most common complication of CD, results from intestinal inflammation and fibrosis. To further explore the functional role of miR‐155 in CD‐associated intestinal fibrosis, we established a colitis model in BALB/C mice and treated these mice with miR‐155 mimic or inhibitor. Overexpression of miR‐155 by mimic or knockdown of miR‐155 inhibitor was confirmed by qRT‐PCR assay (Figure S1A). As shown in Figure 1C, H&E and Masson trichrome staining indicated that TNBS administration successfully developed intestinal fibrosis in mice. Treatment with miR‐155 mimic resulted in extensive intestinal fibrosis and collagen deposition, whereas miR‐155 inhibitor remarkedly attenuated TNBS‐induced fibrosis (Figure 1C). We next examined the expression of fibrosis‐related markers α‐SMA and Collagen I. qRT‐PCR and Western blot analysis showed that overexpression of miR‐155 apparently induced the levels of fibrosis markers, whereas inhibition of miR‐155 could efficiently abrogate this induction (Figure 1D,E). Consistent with these findings, the accumulation of α‐SMA and Collagen I was visualized in miR‐155 mimic‐treated mice with TNBS‐induced colitis by immunofluorescence staining (Figure 1F).

### 3.3 HBP1 was identified as a direct target of MiR‐155

**FIGURE 2:**
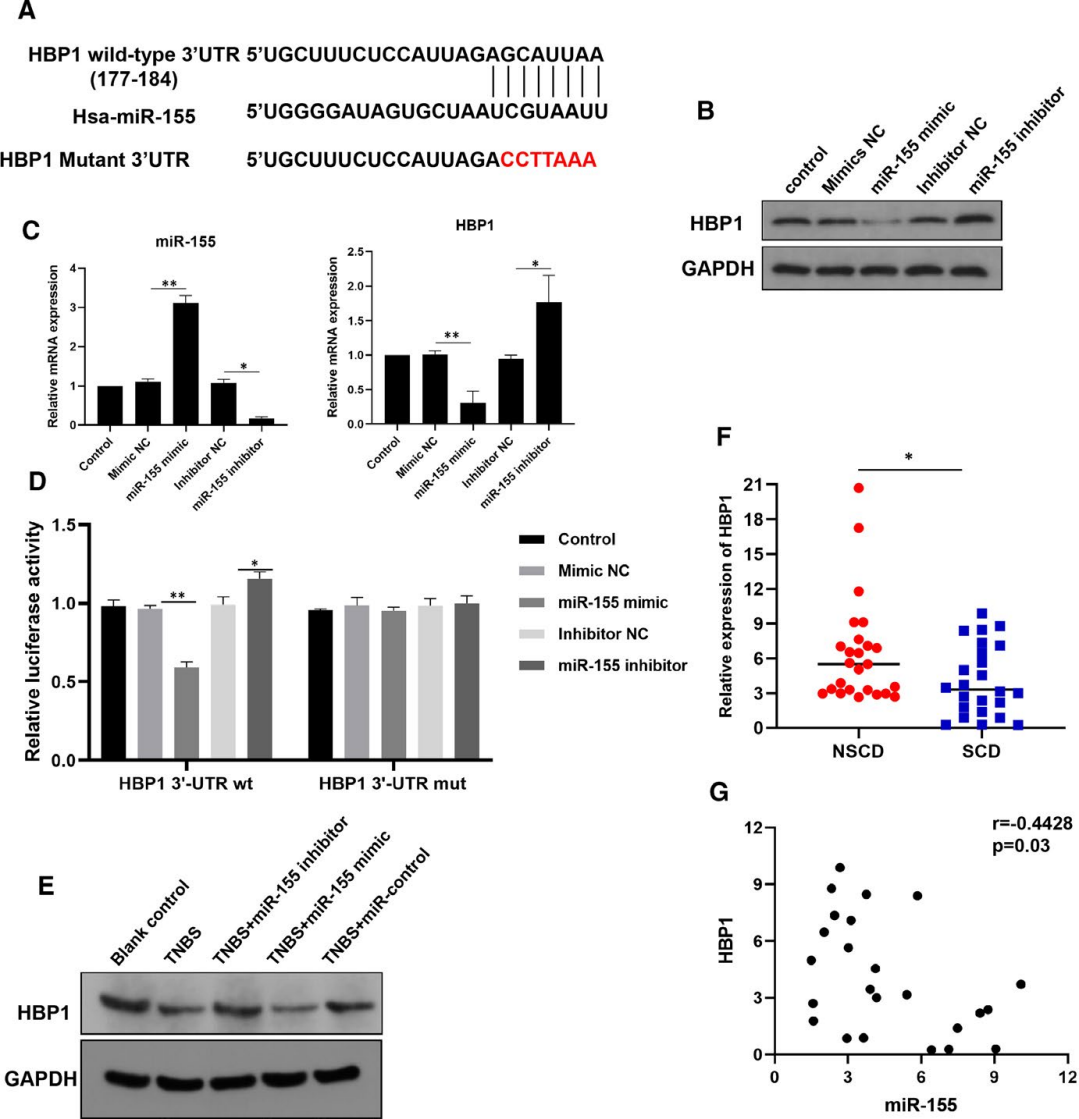
MiR‐155 directly targets and inhibits HBP1. A, The schematic representation of miR‐155 gene promoter with the putative binding sequences and mutation in the 3′UTR of HBP1. B, C, Western blot (B) and qRT‐PCR (C) analysis were performed to detect HBP1 expression in human colonic fibroblast CCD‐18Co cells transfected with miR‐155 mimic or inhibitor. D, Luciferase reporter assay was used to determine miR‐155 direct targeting the HBP1 3′UTR in cells following co‐transfection with wild‐type or mutant HBP1 3′‐UTR reported together with control, miR‐155 mimic or inhibitor. E, Western blot analysis was used to detect HBP1 protein expression in TNBS‐induced colitis with fibrosis mouse model after treatment with miR‐155 mimic or inhibitor. F, The qRT‐PCR analysis was used to detect the expression of HBP1 in CD patients with or without intestinal stricture. G, Pearson's correlation analysis was performed to predict the relationship between miR‐155 and HBP1 in SCD patients. *P < 0.05, **P < 0.01, ***P < 0.001

To figure out the underlying mechanism of miR‐155 on facilitating intestinal fibrosis, we performed the bioinformatic analysis to identify a binding site for miR‐155 with HBP1 3′‐UTR region (Figure 2A). The protein and mRNA expression of HBP1 was further determined in CCD‐18Co cells transfected with miR‐155 mimic or inhibitor. Obviously, overexpression of miR‐155 significantly suppressed the expression of HBP1, whereas HBP1 was comparatively increased after the inhibition of miR‐155 (Figure 2B,C). To determine the binding situation of miR‐155 on HBP1, the wild and mutated 3′‐UTR sequences of HBP1 were designed and cloned into a luciferase expression vector. We found that miR‐155 mimic significantly inhibited the wild‐type HBP1 3′‐UTR activity, whereas there was no inhibitory effect of miR‐155 on mutant HBP1 3′UTR activity. These results were further supported by the observation that miR‐155 inhibitor up‐regulated the 3′‐UTR luciferase activity of wild‐type HBP1 (Figure 2D). Consistent with the in vitro results, miR‐155 was demonstrated to suppress the protein expression of HBP1 in the TNBS‐induced colitis mouse model, as compared to the control group (Figure 2E). These data confirmed that HBP1 is indeed a direct target of miR‐155.

Next, the mRNA levels of HBP1 in CD patients were detected. There was no significant difference on the expression of HBP1 in CD patients compared to health controls (Figure S1B). However, HBP1 was down‐regulated in the patients with intestinal stricture, compared to CD patients without intestinal stricture (Figure 2F). Given that miR‐155 was increased in CD patients with intestinal stricture, Pearson's correlation analysis was performed to predict the relationship between HBP1 and miR‐155. It has been shown that the levels of miR‐155 were negatively correlated with the levels of HBP1 in SCD patients (Figure 2G).

### 3.4 Knockdown of HBP1 promoted intestinal fibrosis

**FIGURE 3:**
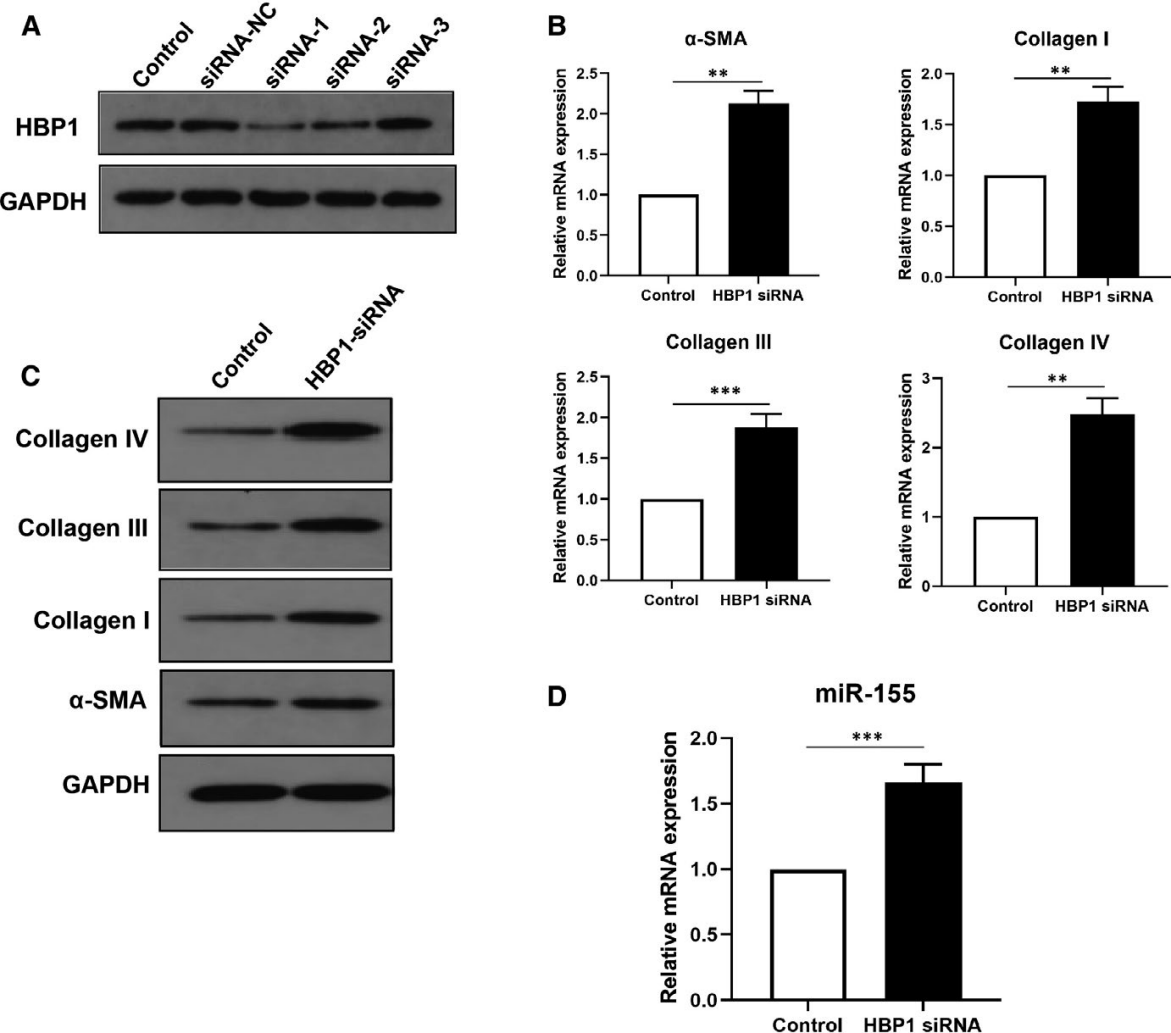
Knockdown of HBP1 promoted intestinal fibrosis. A, Western blot analysis was performed to verify the expression of HBP1 in CCD‐18Co cells transfected with HBP1 siRNAs. B, C, qRT‐PCR and Western blot analysis were used to assess the mRNA (B) and protein (C) expression of fibrosis‐related markers α‐SMA, Collagen I, III and IV in cells transfected with HBP1 siRNA. D, The miR‐155 levels were detected by qRT‐PCR assay after transfection with HBP1 siRNA. *P < 0.05, **P < 0.01, ***P < 0.001

To determine whether HBP1 is involved in the progression of colitis‐related intestinal fibrosis, three small‐interfering RNAs (siRNAs) were used to silence HBP1 expression in CCD‐18Co cells (Figure S1C and Figure 3A). It has been shown that HBP1 siRNA1 significantly knock down the levels of HBP1. Hence, it was used for subsequent experiments. Next, fibrosis‐associated markers were examined after HBP1 siRNA was transfected into CCD‐18Co cells. As expected, knockdown of HBP1 increased the mRNA and protein levels of fibrosis markers including α‐SMA, Collagen I, Collagen III and Collagen IV (Figure 3B,C). Interestingly, we found that HBP1 in turn negatively regulated the expression of miR‐155 (Figure 3D). Collectively, these data suggested that miR‐155 promoted intestinal fibrosis by targeting suppression of HBP1.

### 3.5 MiR‐155 targets HBP1 to activate Wnt/β‐catenin signalling pathway

**FIGURE 4:**
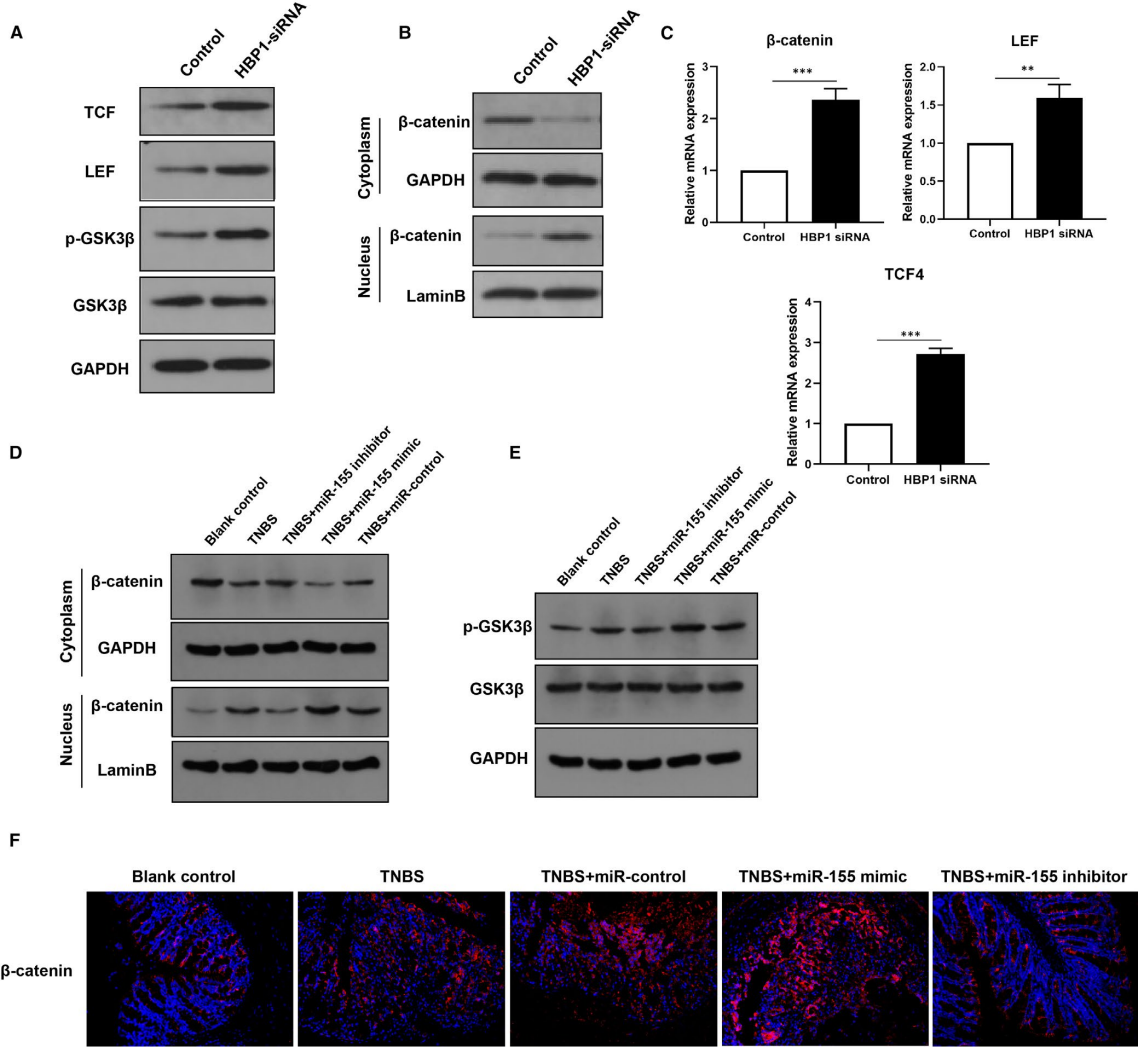
MiR‐155 targets HBP1 to activate Wnt/β‐catenin signalling pathway. A, The protein expression of the components in Wnt/β‐catenin signalling pathway including TCF4, LEF, phosphorylated and total GSK3β by Western blot analysis in cells transfected with HBP1 siRNA. B, After treated with HBP1 siRNA, the cytoplasmic and nuclear fraction lysates were used to analysis the translocation of β‐catenin by immunoblotting. C, The qRT‐PCR analysis was used to determine mRNA levels of the components in Wnt/β‐catenin signalling pathway. D, After administrated with miR‐155 control, mimic or inhibitor in TNBS‐induced colitis mouse model, the intestine tissues were collected to detect the expression of nuclear and cytoplasmic β‐catenin. E, The phosphorylation of GSK3β was assessed by immunoblotting in the intestine tissues from colitis mouse models following the treatment with miR‐155 control, mimic or inhibitor. F, Immunofluorescence assay was performed to determine the expression of β‐catenin in the intestine tissues from animal models following TNBS treatment in combination with miR‐155 control, mimic or inhibitor. *P < 0.05, **P < 0.01, ***P < 0.001

We next investigated the underlying mechanism by which miR‐155 and HBP1 mediate intestinal fibrosis. HBP1 has been identified as a negative regulate Wnt/β‐catenin signalling pathway. To determine the effects of miR‐155 and HBP1 on Wnt/β‐catenin signalling, we first examined several core components of Wnt/β‐catenin signalling by Western blot analysis after HBP1 siRNA transfected into CCD‐18Co cells. As shown in Figure 4A, knockdown of HBP1 resulted in a increase of phosphorylated GSK3β levels and increased expression levels of TCF4 and LEF transcriptional factors (Figure 4A). Furthermore, treatment with HBP1 siRNA caused the accumulation of β‐catenin in the nucleus (Figure 4B). In support of the data of Western blot analysis, silencing HBP1 increased the mRNA levels of β‐catenin, TCF4 and LEF (Figure 4C). These results suggested that HBP1 might contribute to the inhibition of Wnt/β‐catenin signalling pathway.

Then, we explored the regulation of miR‐155 on the Wnt/β‐catenin signalling pathway. Before TNBS‐induced colitis, mice received an intravenous injection of specific miR‐155 mimic or inhibitor oligonucleotide. Similarly, compared with the scrambled control group, miR‐155 mimic treatment resulted in nuclear accumulation of β‐catenin, whereas miR‐155 inhibitor promoted its cytoplasmic localization (Figure 4D). The phosphorylated GSK3β levels were also increased in miR‐155 mimic‐treated mice (Figure 4E). To further support the effect of miR‐155 on Wnt signalling, immunofluorescence staining was performed for β‐catenin expression. Consistently, miR‐155 remarkedly enhanced β‐catenin expression (Figure 4F). Taken together, these findings suggested that miR‐155 targets HBP1 to activate the Wnt/β‐catenin signalling pathway.

### 3.6 The activation of Wnt/β‐catenin signalling pathway is required for miR‐155‐induced intestinal fibrosis

**FIGURE 5:**
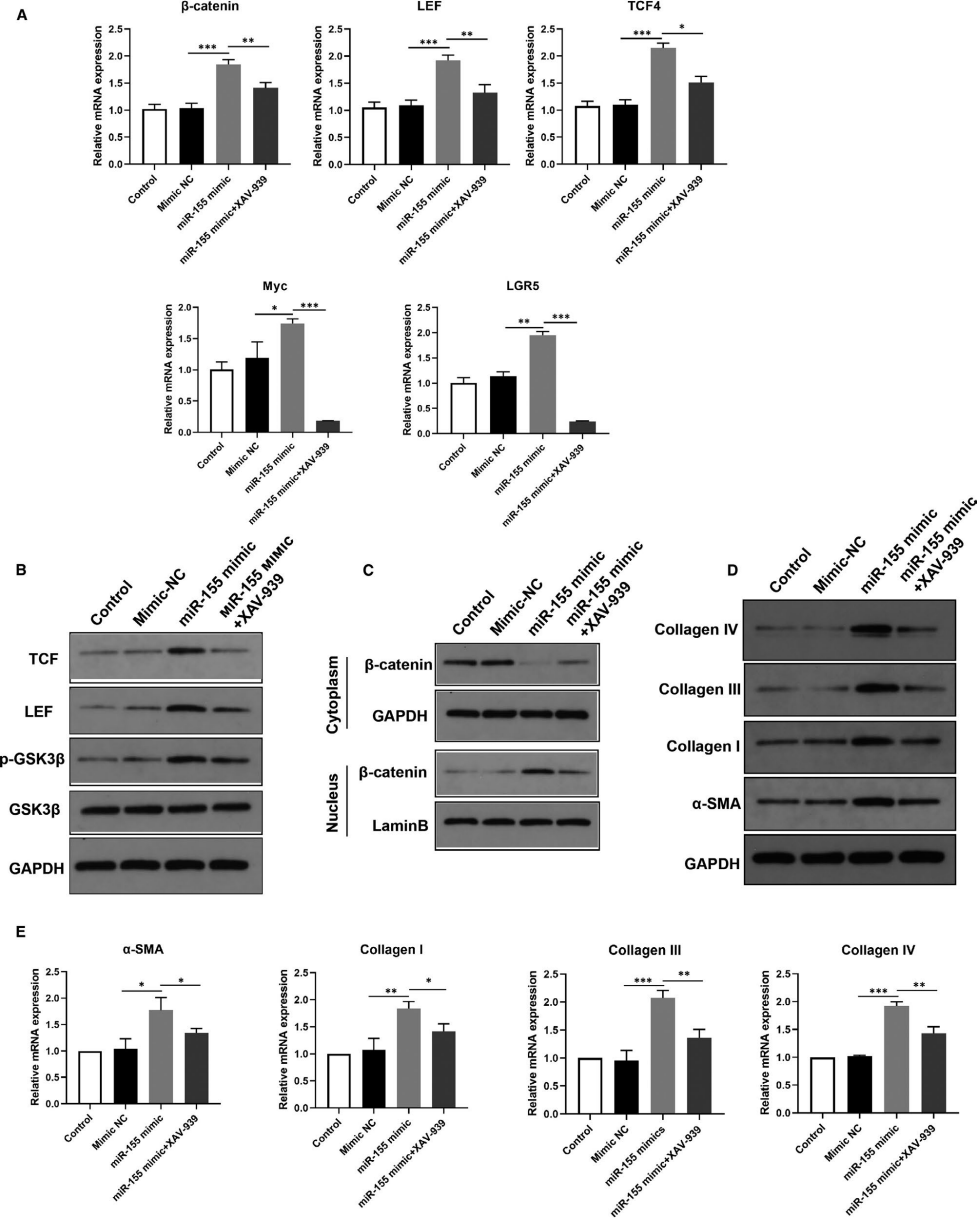
The activation of Wnt/β‐catenin signalling pathway is required for miR‐155‐induced intestinal fibrosis. A, The mRNA levels of β‐catenin, LEF, TCF4, Myc and LGR5 were detected in CCD‐18Co cells treated with miR‐155 mimic in combination with the β‐catenin inhibitor XAV‐939. B, The protein levels of LEF, TCF and phosphorylated GSK3β were assessed by immunoblotting after treatment with miR‐155 mimic and XAV939. C, After treatment with miR‐155 mimic and XAV939, the cytoplasmic and nuclear fraction lysates were used to analyse the translocation of β‐catenin by immunoblotting. D, E, Western blot and qRT‐PCR assays were used to determine the protein and mRNA levels of fibrosis markers α‐SMA, Collagen I, III and IV in cells treated with miR‐155 mimic in combination with XAV939. *P < 0.05, **P < 0.01, ***P < 0.001

To determine whether the Wnt/β‐catenin signalling is involved in the regulation of miR‐155 on intestinal fibrosis, the human colonic CCD‐18Co fibroblast cells were transfected with miR‐155 and then treated with XAV939, a Wnt/β‐catenin inhibitor. MiR‐155 overexpression up‐regulated the mRNA levels of β‐catenin, LEF and TCF4 in Wnt signalling. Likewise, the target genes of Wnt signalling including Myc and LGR5 were increased following miR‐155 mimic treatment. Such changes were reversed by treatment with XAV‐939 (Figure 5A). Similarly, XAV‐939 treatment decreased the phosphorylation of GSK3β (Figure 5B). Furthermore, miR‐155 induced nuclear accumulation of β‐catenin, which partially translocated into the cytoplasm following XAV‐939 treatment (Figure 5C). Next, we examined the expression of fibrotic markers. As respected, the overexpression of miR‐155 significantly up‐regulated the protein and mRNA levels of α‐SMA, Collagen I, Collagen III and IV, which were reversed these effects by the inhibition of β‐catenin with XAV‐939 (Figure 5D,E).

## 4 DISCUSSION

Intestinal fibrosis is the most common complication of CD that can result in stricture formation. The role of miR‐155 in the progression of intestinal fibrosis and the associated molecular mechanism is still not clear. In this study, we found that the miR‐155 levels were increased in CD patients with intestinal stricture compared to NSCD patients. We further determined that the overexpression of miR‐155 significantly aggravated intestinal fibrosis in the TNBS‐induced colitis mouse model, whereas inhibition of miR‐155 exerted the opposite effects. Taken together, our data suggest the pathogenic role of miR‐155 in promoting the development of intestinal fibrosis in CD.

Emerging evidence has indicated that the dysregulation of many miRNAs is involved in colitis‐associated intestinal fibrosis.^31^ miR‐200b has been reported to inhibit the process of intestinal fibrosis by suppressing ZEB1 and ZEB2.^32^, ^33^ It has been found that the miR‐19b family is involved in CD fibrosis.^34^ MiR‐155 is a multifunctional miRNA with an inflammatory response regulator and oncogenic roles.^35^ Fibrosis is a pathophysiological feature with an excessive accumulation of ECM, which results from long‐term chronic inflammation. In the present study, miR‐155 is defined to promote intestinal fibrosis progression. These findings are consistent with previous studies that miR‐155 functions as a pro‐inflammatory mediator.^16^, ^36^

By interacting with target sites located in 3′‐UTR, miRNA directly suppresses post‐transcriptional levels of their target mRNAs by degradation or translational inhibition. Depending on the biological and clinical role of their target mRNAs, miRNAs show different pathological functions. Accumulating data show that miR‐155 can directly target and inhibit many genes, such as BCL2, PDCD4, SOX and SHIPI genes, which are implicated in diverse cellular biological process including inflammatory response, cell proliferation, apoptosis and chemoresistance.^35^, ^37^, ^38^, ^39^ It has been found that SHIPI is a direct target of miR‐155 and inhibited by the overexpression of miR‐155, therefore promoting the induction of pro‐inflammatory cytokines, such as TNF‐α and IL‐1β.^38^, ^40^ In addition, miR‐155 has been demonstrated to directly inhibit the expression of PDCD4 and further regulate cell proliferation in Tongue cancer.^39^ In this study, our data showed that miR‐155 directly targets HBP1 mRNA and blocks its expression by Western blotting, qRT‐PCR and luciferase reporter assays. Furthermore, knockdown of HBP1 predominantly increased the expression of fibrosis markers including α‐SMA, Collagen I, Collagen III and IV. Our results are also consistent with the previous studies reporting that HBP1 acts as a primary target of miR‐155. Tian et al indicated that miR‐155 interacted with and repressed 3′‐UTR of HBP1 in atherogenesis. Mechanistically, it has been reported that miR‐155 promoted foam cell formation in oxidized low‐density lipoprotein (oxLDL)‐induced macrophages by inhibition of HBP1.^27^ Wan et al^41^ also demonstrated the oncogenic role of miR‐155 in colorectal carcinoma by suppressing the expression of HBP1. Therefore, these findings suggest that HBP1 as a direct target of miR‐155 plays a crucial role in the regulation of miR‐155 on multiple diseases.

The Wnt/β‐catenin signalling pathway is important for many essential processes in embryonic development and adult tissue homeostasis. The destruction complex composed of GSK3β, APC and AXIN trigger the ubiquitination and degradation of cytosolic β‐catenin.^42^ The phosphorylation of GSK3β leads to the activation and accumulation of β‐catenin, which then translocates into the nucleus and binds to TCF/LEF transcriptional factors.^43^ As a result, the expression of downstream target genes such as Myc, cyclin D1 and LGR5 is up‐regulated. Numerous evidence has shown the important interaction between miRNAs and the Wnt/β‐catenin signalling pathway. MiR‐4476 has been reported to directly target the essential component of Wnt signalling APC to increase the activity of β‐catenin.^44^ MiR‐155‐5p, derived from the miR‐155, is found to regulate Wnt/β‐catenin signalling pathway in familial adenomatous polyposis by targeting the components AXIN1 and TCF4.^45^ In this study, we revealed that miR‐155 inhibits the expression of HBP1 that is a well‐known negative regulator of the Wnt/β‐catenin signalling pathway. More importantly, our in vivo and in vitro studies indicated that overexpression of miR‐155 up‐regulates phosphorylated GSK3β, LEF and TCF4 levels, leading to the accumulation of nuclear β‐catenin. Together, these results support that miR‐155 activates the Wnt/β‐catenin signalling pathway by directly targeting HBP1.

Recent studies have investigated the regulation of miR‐155 on the Wnt/β‐catenin signalling pathway. Liu et al^13^ indicated that miR‐155 could promote the invasion ability of colon cancer cell through activating β‐catenin. Additionally, it has been found that the Wnt/β‐catenin signalling pathway may be involved in the regulation of miR‐155 on the depression‐like behaviours of mice.^46^ However, the role of Wnt/β‐catenin signalling in miR‐155‐induced CD‐associated intestinal fibrosis has not been elucidated yet. Our results indicated that miR‐155 led to the up‐regulation of fibrosis‐related markers, α‐SMA, Collagen I, Collagen III and IV in human fibroblast cells. These effects were suppressed by treatment with XAV‐939, a β‐catenin inhibitor. Of note, the aberrant regulation of Wnt/β‐catenin signalling pathway is associated with a broad range of pathologies, including cancers and fibrosis disorders. The activation of Wnt/β‐catenin signalling appears to induce the markers of injury and fibrosis in a variety of different fibrotic diseases, including liver fibrosis, pulmonary fibrosis^47^ and kidney firbosis.^48^ It has been proposed that the altered activity of β‐catenin could result in the activation of hepatic stellate cells, which is responsible for liver fibrosis.^49^ Our results suggested that miR‐155 induces intestinal fibrosis through activation of the Wnt/β‐catenin signalling pathway. Future investigations are warranted to confirm the role of Wnt/β‐catenin signalling in colitis‐related intestinal fibrosis in the animal model and human tissues.

**FIGURE 6:**
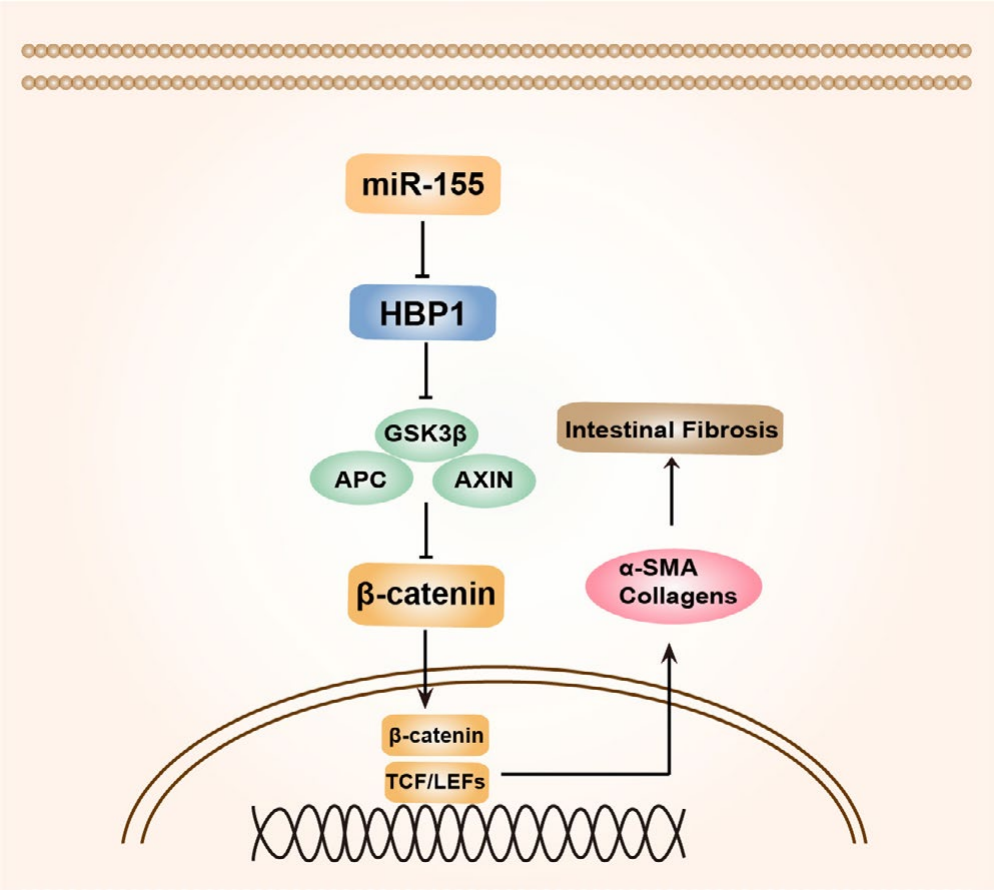
The proposed mechanism of miR‐155‐mediated colitis‐associated intestinal fibrosis. The miR‐155 could directly target and repress the expression of HBP1, which negatively regulates the Wnt/β‐catenin signalling pathway. As a result, miR‐155 activates colitis disease‐related intestinal fibrosis by regulation of the HBP1/Wnt/β‐catenin pathway

In summary, we proposed and demonstrated a working model to illustrate the role of miR‐155 in promoting CD‐associated intestinal fibrosis (Figure 6). We found that miR‐155 activates the Wnt/β‐catenin signalling by directly targeting its negative regulator HBP1. The findings from this study may unveil mechanistic insight into why miR‐155 induces intestinal fibrosis, thereby providing a potential therapeutic target for the treatment of colitis‐related intestinal strictures.

## 5 ETHICAL APPROVAL STATEMENT

The biopsy specimens and all animal experiments were approved by the ethics committees of the First Affiliated Hospital of Nanchang University.

## CONFLICT OF INTEREST

The authors declare that they have no competing interests.

## AUTHOR CONTRIBUTIONS

**Zhenhua Zhu:** Conceptualization (equal); Funding acquisition (equal); Project administration (equal); Supervision (equal); Writing‐review & editing (equal). **Nianshuang Li:** Data curation (equal); Funding acquisition (equal); Methodology (equal); Writing‐original draft (equal); Writing‐review & editing (equal). **Yaobin Ouyang:** Methodology (equal); Resources (equal); Writing‐review & editing (equal). **Xinbo Xu:** Data curation (equal); Methodology (equal); Resources (equal); Validation (equal). **Zhenxiang Yuan:** Methodology (equal); Resources (equal); Software (equal). **Chuanquan Liu:** Methodology (equal); Validation (equal).

## Supporting information

Fig S1

Table S1‐S3

## Data Availability

The data that support the findings of this study are available from the corresponding author upon reasonable request.
