# Supplementary figures and images for: MiR‐155 promotes colitis‐associated intestinal fibrosis by targeting HBP1/Wnt/β‐catenin signalling pathway
Source: J Cell Mol Med. 2021 Mar 26;25(10):4765–75. doi: 10.1111/jcmm.16445 (PMC8107084; doi:10.1111/jcmm.16445)

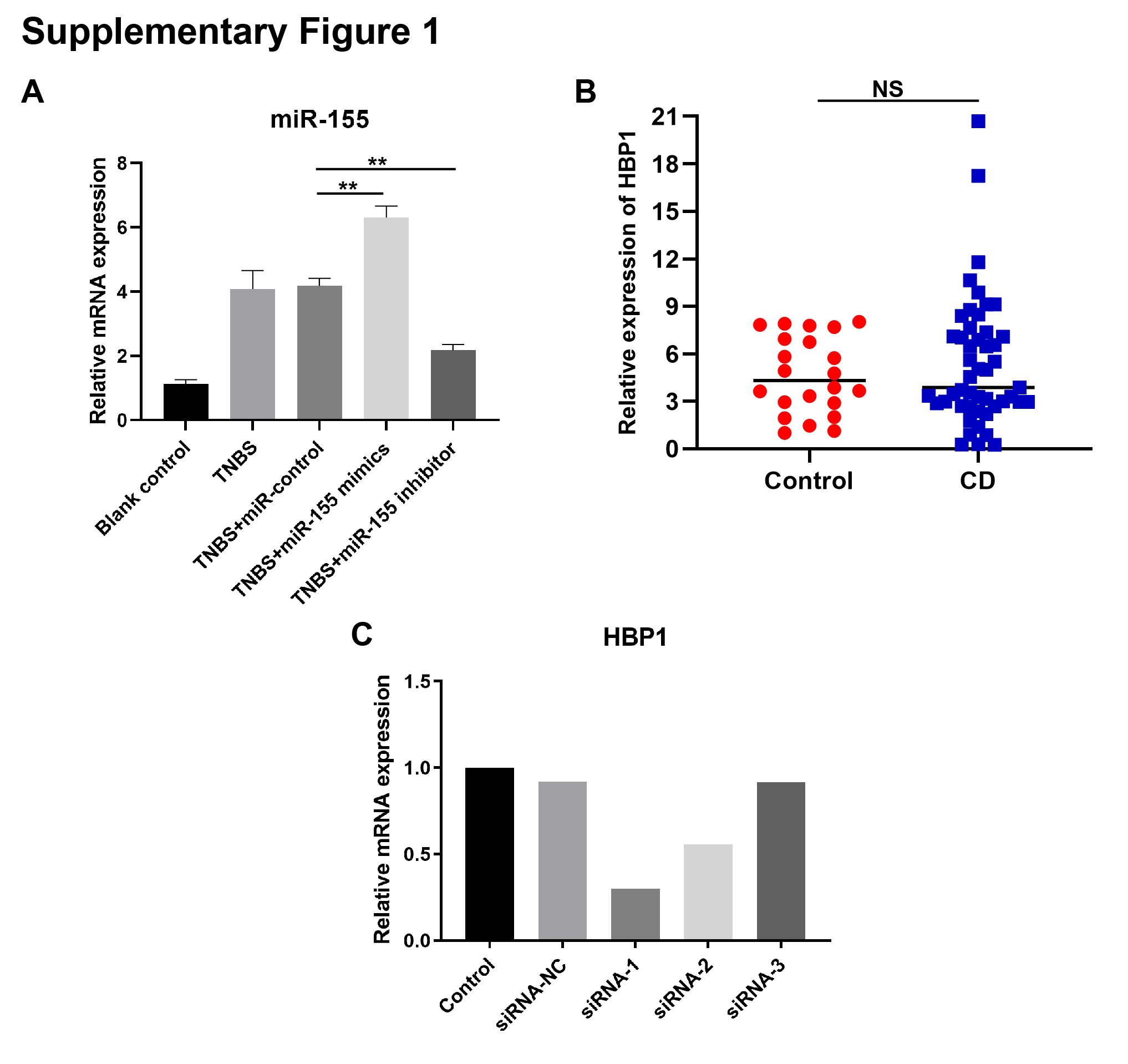

Supplement: Supplementary file 1 — Fig S1 [file JCMM-25-4765-s001.jpg]
