# Supplementary material for: MiR‐155 promotes colitis‐associated intestinal fibrosis by targeting HBP1/Wnt/β‐catenin signalling pathway
Source: J Cell Mol Med. 2021 Mar 26;25(10):4765–75. doi: 10.1111/jcmm.16445 (PMC8107084; doi:10.1111/jcmm.16445)
Supplement: Supplementary file 2 — Table S1‐S3 [file JCMM-25-4765-s002.docx]

**Table S1: Sequences of siRNAs used in this study.**

| siRNA primer | siRNA sequence |
| --- | --- |
| HBP1 siRNA-1 Forward | 5’-GCUGCAGUGUAAUGAGAAUTT-3’ |
| HBP1 siRNA-1 Reverse | 5’-AUUCUCAUUACACUGCAGCTT-3’ |
| HBP1 siRNA-2 Forward | 5’-CCACCAGUGUCCUCUUCUUTT-3’ |
| HBP1 siRNA-2 Reverse | 5’-AAGAAGAGGACACUGGUGGTT-3’ |
| HBP1 siRNA-3 Forward | 5’-GGUACAGUAGAAGAUGGUUTT-3’ |
| HBP1 siRNA-3 Reverse | 5’-AACCAUCUUCUACUGUACCTT-3’ |
| NC Forward | 5’-UUCUCCGAACGUGUCACGUTT-3’ |
| NC Reverse | 5’-ACGUGACACGUUCGGAGAATT-3’ |

**Table S2: Antibodies used in this study.**

| **Antigen detected** | **Cat** | **Source** |
| --- | --- | --- |
| GAPDH | AB-P-R 001 | Xianzhi biology, Hangzhou, China |
| Lamin B | BA1228 | Boster Biological Technology, Wuhan, China |
| HBP1 | 11746-1-AP | Proteintech, Wuhan, China |
| β-catenin | 17565-1-AP | Proteintech, Wuhan, China |
| GSK3β | 22104-1-AP | Proteintech, Wuhan, China |
| P-GSK3β (ser9) | #9322 | CST, Beverly, MA, USA |
| LEF | 14972-1-AP | Proteintech, Wuhan, China |
| TCF | 22337-1-AP | Proteintech, Wuhan, China |
| α-SMA | 55135-1-AP | Proteintech, Wuhan, China |
| Collagen I | 14695-1-AP | Proteintech, Wuhan, China |
| Collagen III | 22734-1-AP | Proteintech, Wuhan, China |
| Collagen IV | ab6586 | Abcam, Cambridge, UK |

**Table S3: Sequences of primers used in this study.**

| **Gene primer** | **Sequence** |
| --- | --- |
| Human GAPDH Forward | 5‘-TCAAGAAGGTGGTGAAGCAGG-3’ |
| Human GAPDH Reverse | 5‘-TCAAAGGTGGAGGAGTGGGT-3’ |
| Human HBP1 Forward | 5‘-GGAGGAAACACCAGTAAGACACGAA-3’ |
| Human HBP1 Reverse | 5‘-AAAAACAGTGCCAGACAGTTGAAGG-3’ |
| Human β-catenin Forward | 5‘-CTGCCAAGTGGGTGGTATAGAGG-3’ |
| Human β-catenin Reverse | 5‘-CAGTGGGATGGTGGGTGTAAGAG-3’ |
| Human LEF Forward | 5‘-GATGCCAAATATGAATAACGACCCA-3’ |
| Human LEF Reverse | 5‘-CAACACCACCCGGAGACAA-3’ |
| Human TCF4 Forward | 5‘-TCCATCAGCAAGCACTGCCGACTA-3’ |
| Human TCF4 Reverse | 5‘-TTGCCCAACATTCCTGCATAGCC-3’ |
| Human α-SMA Forward | 5‘-TCATGGTCGGTATGGGTCAG-3’ |
| Human α-SMA Reverse | 5‘-CGTTGTAGAAGGTGTGGTGC-3’ |
| Human Collagen I Forward | 5‘-CCTTATGCCTAGCAACATGCCAATC-3’ |
| Human Collagen I Reverse | 5‘-GAGCAGCAAAGTTCCCACCGAGA-3’ |
| Human Collagen III Forward | 5‘-AAAAGGGGAGCTGGCTACTT-3’ |
| Human Collagen III Reverse | 5‘-TGGGTTGGGGCAGTCTAATT-3’ |
| Human Collagen IV Forward | 5‘-ATTACACGCCACAGCCAGACAA -3’ |
| Human Collagen IV Reverse | 5‘-GCATGGTACTAAAGCGACGAAGG-3’ |
| Human MYC Forward | 5‘-GGCTCCTGGCAAAAGGTCA-3’ |
| Human MYC Reverse | 5‘-CTGCGTAGTTGTGCTGATGT-3’ |
| Human LGR5 Forward | 5‘-CTCCCAGGTCTGGTGTGTTG-3’ |
| Human LGR5 Reverse | 5‘-GAGGTCTAGGTAGGAGGTGAAG-3’ |
| U6 Forward | 5‘-CGCTTCGGCAGCACATATAC-3’ |
| U6 Reverse | 5‘-AAATATGGAACGCTTCACGA-3’ |
| Human HBP1-3’UTR-mut Forward | 5‘-TTCTCCATTAGACCTTAAAGCTAAAACTATCAACATT-3’ |
| Human HBP1-3UTR-mut Reverse | 5‘-GATAGTTTTAGCTTTAAGGTCTAATGGAGAAAGCAAAT -3’ |
| Human-miR-155 loop primer | 5‘-GTCGTATCCAGTGCAGGGTCCGAGGTATTCGCACTGGA TACGACACCCCTAT-3’ |
| Human-miR-155 F primer | 5‘-TGCGCTTAATGCTAATCGTGATA-3’ |
| Human-miR-155 R primer | 5‘-CCAGTGCAGGGTCCGAGGTATT-3’ |
| Mouse GAPDH Forward | 5‘-ATGGGTGTGAACCACGAGA-3’ |
| Mouse GAPDH Reverse | 5‘-CAGGGATGATGTTCTGGGCA-3’ |
| Mouse α-SMA Forward | 5‘-CAGCGGGCATCCACGAAACC-3’ |
| Mouse α-SMA Reverse | 5‘-CTTCGTCGTATTCCTGTTTG-3’ |
| Mouse Collagen I Forward | 5‘-CTGACTGGAAGAGCGGAGAG-3’ |
| Mouse Collagen I Reverse | 5‘-GACGGCTGAGTAGGGAACAC-3’ |
| Mouse HBP1 Forward | 5‘-AAGATGAGGTGGACTGGCTAA-3’ |
| Mouse HBP1 Reverse | 5‘-TTGAAGAAGAGGACACTGGT-3’ |
| Mouse miR-155 loop primer | 5‘-GTCGTATCCAGTGCAGGGTCCGAGGTATTCGCACTGGA TACGACACCCC-3’ |
| Mouse miR-155 F primer | 5‘-TGCGCTTAATGCTAATTGTGATA-3’ |
| Mouse miR-155 R primer | 5‘-CCAGTGCAGGGTCCGAGGTATT-3’ |
